# Evaluation of the brain-penetrant microtubule-stabilizing agent, dictyostatin, in the PS19 tau transgenic mouse model of tauopathy

**DOI:** 10.1186/s40478-016-0378-4

**Published:** 2016-09-29

**Authors:** Vishruti Makani, Bin Zhang, Heeoon Han, Yuemang Yao, Pierrik Lassalas, Kevin Lou, Ian Paterson, Virginia M. Y. Lee, John Q. Trojanowski, Carlo Ballatore, Amos B. Smith, Kurt R. Brunden

**Affiliations:** 1 Center for Neurodegenerative Disease Research, Perelman School of Medicine, University of Pennsylvania, 3600 Spruce St., Maloney 3, Philadelphia, PA 19104 USA; 2 Department of Chemistry, School of Arts and Sciences, University of Pennsylvania, Philadelphia, PA 19104 USA; 3 Department of Chemistry, University of Cambridge, Cambridge, CB2 1EW UK

**Keywords:** Alzheimer’s, Drug, Microtubule, Mouse, Pathology, Tauopathy, Transgenic

## Abstract

Neurodegenerative disorders referred to as tauopathies, which includes Alzheimer’s disease (AD), are characterized by insoluble deposits of the tau protein within neuron cell bodies and dendritic processes in the brain. Tau is normally associated with microtubules (MTs) in axons, where it provides MT stabilization and may modulate axonal transport. However, tau becomes hyperphosphorylated and dissociates from MTs in tauopathies, with evidence of reduced MT stability and defective axonal transport. This has led to the hypothesis that MT-stabilizing drugs may have potential for the treatment of tauopathies. Prior studies demonstrated that the brain-penetrant MT-stabilizing drug, epothilone D, had salutary effects in transgenic (Tg) mouse models of tauopathy, improving MT density and axonal transport, while reducing axonal dystrophy. Moreover, epothilone D enhanced cognitive performance and decreased hippocampal neuron loss, with evidence of reduced tau pathology. To date, epothilone D has been the only non-peptide small molecule MT-stabilizing agent to be evaluated in Tg tau mice. Herein, we demonstrate the efficacy of another small molecule brain-penetrant MT-stabilizing agent, dictyostatin, in the PS19 tau Tg mouse model. Although dictyostatin was poorly tolerated at once-weekly doses of 1 mg/kg or 0.3 mg/kg, likely due to gastrointestinal (GI) complications, a dictyostatin dose of 0.1 mg/kg was better tolerated, such that the majority of 6-month old PS19 mice, which harbor a moderate level of brain tau pathology, completed a 3-month dosing study without evidence of significant body weight loss. Importantly, as previously observed with epothilone D, the dictyostatin-treated PS19 mice displayed improved MT density and reduced axonal dystrophy, with a reduction of tau pathology and a trend toward increased hippocampal neuron survival relative to vehicle-treated PS19 mice. Thus, despite evidence of dose-limiting peripheral side effects, the observed positive brain outcomes in dictyostatin-treated aged PS19 mice reinforces the concept that MT-stabilizing compounds have significant potential for the treatment of tauopathies.

## Introduction

Neurodegenerative tauopathies, a group of diseases including Alzheimer’s disease (AD), frontotemporal lobar degeneration (FTLD), progressive supranuclear palsy (PSP), corticobasal degeneration (CBD) and Pick’s disease, are characterized by the presence of inclusions within neurons comprised of the microtubule (MT)-binding protein, tau [4, 25, 33]. These tau deposits, referred to as neurofibrillary tangles when found within neuronal soma and neuritic threads when localized to dendrites, are thought to lead to the neuron loss that is characteristic of all tauopathies. In fact, there is a strong correlation between the density of tau brain pathology and cognitive status in AD [3, 23, 44] and importantly, tau mutations can cause inherited forms of FTLD [27, 28].

**Fig. 1:**
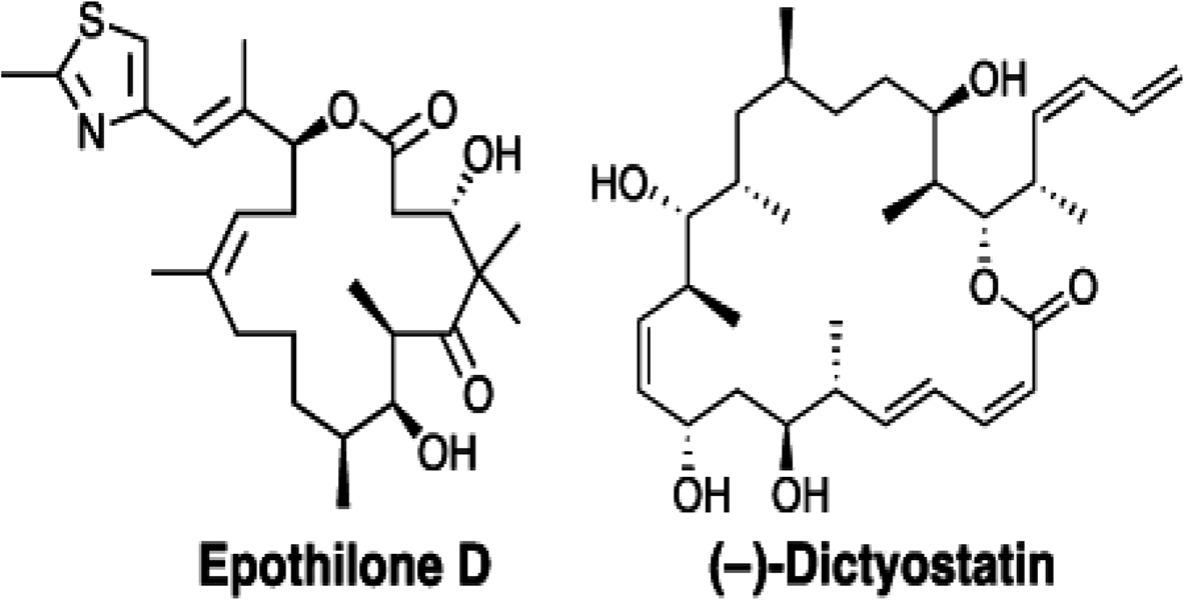
Structures of epothilone D and dictyostatin

Tau is a MT-binding protein in neurons, where it appears to stabilize MT structure [22, 24] and perhaps also play a role in regulating the MT-binding of motor proteins involved in axonal transport [21, 41, 42]. In tauopathies, tau becomes hyperphosphorylated due to an incompletely understood shift in the activity of kinases and/or phosphatases, with a resulting dissociation of tau from MTs [1, 2, 9, 36, 43]. Hyperphosphorylation may also facilitate the misfolding and assembly of tau into fibrils that form inclusions [2, 37]. The neurodegeneration that is associated with tau inclusions is thought to result from gain-of-function toxicities attributable to misfolded tau oligomers and/or fibrils, as well as loss-of-function resulting from the decreased binding of hyperphosphorylated tau to MTs, with a resulting destabilization of MTs and/or impairment of axonal transport [4]. Accordingly, various therapeutic strategies have been suggested to reduce the consequences of tau pathology in neurodegenerative disease [12, 13]. Among these are efforts to compensate for tau loss-of-function through the utilization of MT-stabilizing drugs that could “normalize” MTs and axonal transport in tauopathies. Importantly, there is evidence of MT abnormalities in the AD brain [16, 26], as well as in transgenic mouse models of tauopathy [5, 15, 46]. Moreover, we [15, 46] and others [5] have demonstrated that the brain-penetrant MT-stabilizing agent, epothilone D (Fig. 1), significantly improves behavioral and AD-like brain pathological outcomes in Tg mouse tauopathy models. This includes increased MT density, reduced axonal dystrophy, and improved axonal transport with a salvaging of hippocampal neurons and an apparent reduction of tau pathology. To date, epothilone D is the only non-peptide small molecule MT-stabilizing compound that has been shown to reduce the consequences of tau inclusion formation in Tg mouse models of tauopathy, although the octapeptide NAP (also called davunetide) has been shown to improve outcomes in tau Tg mice through mechanisms [34] that include MT stabilization [20, 35].

Recently we reported on another MT-stabilizing agent, dictyostatin (Fig. 1), which like epothilone D is a natural product that readily enters the brain upon peripheral administration and increases a marker of stable MTs (acetyl-tubulin) in wild-type mice after a single administration at relatively low doses [11]. Interestingly, both dictyostatin and epothilone D show prolonged brain retention after clearance from blood, unusual pharmacokinetic behavior which we postulate might provide a safety and efficacy benefit in the treatment of tauopathies [10, 13]. A long brain half-life permits infrequent dosing (once weekly in prior studies with epothilone D), resulting in clearance of the MT-stabilizing drug from the periphery that potentially minimizes side effects such as neutropenia and peripheral neuropathy that are typically observed with drugs of this class when utilized for the treatment of cancers [6, 17, 18]. Given the similarities in the brain pharmacokinetic and pharmacodynamics properties of epothilone D and dictyostatin, we examine here the latter MT-stabilizing agent in the PS19 tau Tg model of tauopathy [45]. We find that dictyostatin, like epothilone D, provided benefit to the central nervous system (CNS) of PS19 mice, improving MT density and decreasing axonal abnormalities while reducing tau pathology, with a trend towards increased hippocampal neuron survival. However, unlike epothilone D, we observed an unexpected dose-limiting side effect with dictyostatin that led to gastrointestinal (GI) complications and death of some PS19 mice. The totality of these data provide further support for the potential value of small molecule brain-penetrant MT-stabilizing agents in correcting MT and/or axonal transport deficiencies in AD and related tauopathies, but they also emphasize that care will be required in identifying MT-stabilizing drug candidates with the proper balance of efficacy and safety.

## Materials and methods

### Preparation of dictyostatin

Dictyostatin was synthesized as a white solid (26mg, 0.49mmol) according to reported procedures [38] and purified by silica gel flash column chromatography (purity level > 95%, as determined by HPLC and NMR). All the spectral data (1H-NMR, 13C-NMR, HR-Mass) were identical to reported data [38].

### Dictyostatin or vehicle treatment of PS19 and wild-type mice

A transgene encoding the human T34 tau isoform (1N4R) containing the P301S mutation found in FTDP-17, driven by the mouse PrP promoter, was used to create tau Tg mice [45]. These mice were originally derived on a B6/C3H background, and underwent multiple generations of backcrossing to yield the congenic B6 PS19 mice used here. In an initial study, 6-month old male B6 PS19 tau Tg mice received weekly intraperitoneal (ip) injections with vehicle (DMSO), 1.0 mg/kg dictyostatin or 0.3 mg/kg dictyostatin. As clear evidence of intolerance was observed at these doses, a study was initiated in which 6-month old male PS19 mice (*n* = 11 per group) were given weekly ip injections of vehicle or 0.1 mg/kg dictyostatin, or a biweekly dose of 0.3 mg/kg dictyostatin. In addition, age-matched wild-type male B6 mice (*n* = 9) received weekly ip administration of vehicle (DMSO). Animals were monitored for signs of abnormal behavior or distress, and were weighed weekly until the completion of study. After 14 weeks of dosing, surviving animals were sacrificed according to a University of Pennsylvania IACUC-approved protocol, with blood collected for analysis of complete blood cell counts. Major organs were removed for weighing, and brains and optic nerves (ONs) were harvested for further analyses.

### Electron Microscopy (EM) evaluation of ON MT density and axonal dystrophy

Transmission EM was performed on cross-sections of ONs from vehicle- and dictyostatin-treated PS19 mice or vehicle-treated B6 wild-type mice essentially as previously described [15, 46]. Briefly, ONs were post-fixed in 2% glutaraldehyde and 2% paraformaldehyde and then processed for standard EM [47]. Ultrathin ON sections were mounted on EM grids and examined using a JEM1010 electron microscope (Jeol, Peabody, MA) at 80 kV. Cross sections of the entire ON were systematically sampled at 50,000x and 10,000x magnification, with MTs and dystrophic axons counted from multiple fields of 0.035 μm^2^ and 128 μm^2^ respectively, from at least 25 photos per ON. The assessors were masked to the treatment group.

### Complete blood counts

Blood from vehicle- or dictyostatin-treated PS19 mice was analyzed for complete blood cell counts as previously described (Brunden et al., 2010).

### Immunohistochemical analyses

Mice were euthanized by an ip injection of ketamine hydrochloride (1 mg/10 g) and xylazine ((0.1 mg/10 g), followed by intracardial perfusion with 20 mL of PBS. The brains of the mice were then removed and one hemisphere of each brain was processed as described previously [15]. Paraffin embedded brain hemispheres were cut into 6 μm thick sections using a sliding microtome. Immunostained sections that were masked to treatment identification were imaged using a Lamina multilabel slide scanner. For analysis of hippocampal neurons, two matched brain sections (Bregma:−1.70 to −1.82) from vehicle- and dictyostatin-treated PS19 mice were manually annotated around the CA3 region of the hippocampus using HALO (Indica Labs, Corrales, NM) software. To determine neuronal density, sections were stained with the neuron-specific NeuN antibody (1:500 dilution; Millipore) and the end boundary of the CA3 region was defined by drawing a line from the dentate to the start of the CA2 region (blue line in Fig. 6a), also using the demarcation of the end of the CA2 region (black arrow in Fig. 6a) as a landmark. The NeuN-positive CA3 area was quantified using HALO software after threshold adjustment to eliminate background signal. Analysis of tau pathology was performed using two matched brain sections (Bregma:−1.70 to −1.82) from the vehicle- and dictyostatin-treated PS19 mice after immunostaining with the MC1 antibody [31] that recognizes misfolded tau (1:7000 dilution; kind gift from Dr. Peter Davies). The HALO algorithm was set to identify MC1-stained areas, as shown in Fig. 7a, after threshold adjustment to eliminate background signal.

### Analysis of acetylated and AT8-positive insoluble tau

Combined cortex and hippocampus samples (~40–50 mg) from frozen hemispheres from vehicle- and dictyostatin-treated PS19 mice were homogenized in 0.2 ml of RAB high salt buffer (0.1 M MES, 1 mM EGTA, 0.5 mM MgSO_4_, 0.75 M NaCl, 0.02 M NaF, pH 7.0), and the homogenates were centrifuged at 100,000 × g for 30 min at 4^0^ C. The supernatant fraction from each sample was removed and the pellet was re-suspended in 0.1 ml of RAB buffer, followed by another centrifugation as above. The supernatant fraction was combined with that from the first centrifugation, and the pellet was suspended in 0.2 ml of RAB buffer containing 1 M sucrose. The sample was again centrifuged as above, and the supernatant fraction which contained myelin was discarded. The pellet was resuspended in 70 μl of 2% SDS and sonicated, followed by centrifugation at 100,000 × g for 30 min at 22° C. This SDS supernatant fraction was utilized for SDS-PAGE analysis and immunoblotting with a rabbit polyclonal antibody recognizing tau containing an acetyl modification at lysine residue 280 (TauAcK280) [29] or the AT8 monoclonal antibody (ThermoFisher) that recognizes tau that is phosphorylated at serine residue 202 or threonine residue 205. The amount of the RAB-insoluble, SDS-soluble brain sample from each PS19 mouse utilized for SDS-PAGE and immunoblot analysis was determined by comparing the protein concentrations in the corresponding RAB buffer soluble samples, with adjustment of the SDS-soluble sample volumes so that equivalent amounts were loaded based on the RAB-soluble proteins. This method was used since common housekeeping genes typically utilized for internal gel normalization partition into the RAB-soluble brain fraction and are not found in the high salt buffer-insoluble fraction.

### Statistics

The statistical method employed for each data set is described in the Figure legends, as are the group sizes used for the analyses.

## Results

### Tolerability findings with dictyostatin-treated PS19 tau Tg mice

**Fig. 2:**
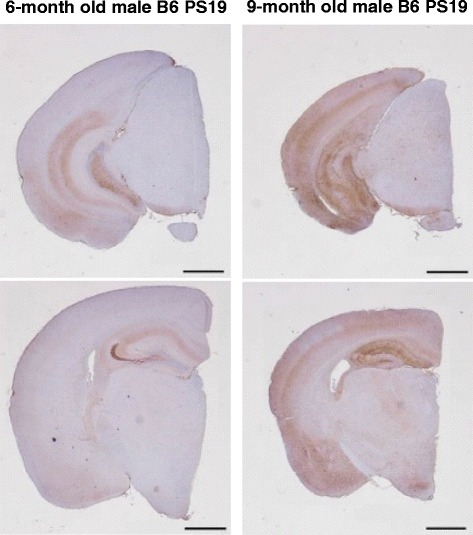
Representative hemisphere brain sections from 6- and 9-month old male B6 PS19 months stained for MC1-positive tau pathology. Bregma-matched sections from male PS19 mice were immunostained with the MC1 antibody and whole hemispheres were imaged. Scale bar = 1 mm

Prior studies from our laboratories demonstrated that a single 5 mg/kg intraperitoneal (ip) administration of dictyostatin (Fig. 1) resulted in a significant increase in brain acetyl-tubulin, a marker of stable MTs [14, 39], in wild-type mice up to 7 days after dosing [11]. Subsequent studies (unpublished) suggested that single doses of 0.5–2 mg/kg of dictyostatin could also elicit a long-lasting increase of brain acetyl-tubulin. We thus began a study in which 6-month old male B6 PS19 Tg mice [45], which express full-length four-repeat tau containing the P301S mutation found in inherited FTLD, were treated weekly with 1 and 0.3 mg/kg of dictyostatin (ip), with the intent of dosing until the mice reached 9 months of age. These doses corresponded to the doses of epothilone D (Fig. 1) that were found to be highly efficacious in previous studies with male PS19 mice [15, 46], although the Tg mice in the prior work were on a B6/C3H hybrid background, whereas in this study PS19 mice were back-crossed to obtain a congenic B6 background that results in somewhat more rapid development of tau pathology. Thus, B6 PS19 mice have a modest amount of tau pathology at 6 months of age which reaches a moderate level my 9 months of age (Fig. 2). Unexpectedly, the dictyostatin-treated PS19 mice in a first small cohort (*n* = 2 per group) showed body weight loss after 3 weeks, with mortality observed in all mice in both of the dictyostatin dose groups after 4 weeks of treatment. An autopsy evaluation of the mice revealed enlargement of the intestines, with an apparent blockage of GI transit, with no other obvious organ abnormalities. This was an unexpected finding, as prior studies with epothilone D did not result in this or other side effects in either PS19 mice with the B6/C3H [15, 46] or B6 background (unpublished).

**Fig. 3:**
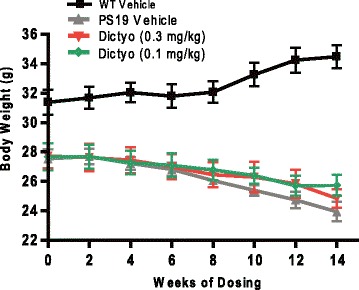
Dictyostatin doses of 0.3 mg/kg biweekly or 0.1 mg/kg weekly did not exacerbate body weight loss in 6-month old PS19 mice. Male 6-month old PS19 mice received either biweekly ip injections of 0.3 mg/kg dictyostatin, weekly injections of 0.1 mg/kg dictyostatin, or weekly injections of vehicle (*n* = 11/treatment). In addition, wild-type male 6-month old B6 mice received weekly injections of vehicle. Body weights were recorded weekly. A total of 6 mice in the 0.3 mg/kg dictyostatin group and 3 mice in the 0.1 mg/kg dictyostatin group died before study completion. Error bars represent SEM

Because of the side effects caused by the weekly 1 and 0.3 mg/kg doses of dictyostatin in PS19 mice, another study was initiated in which 6-month old male B6 PS19 mice received weekly ip administration of vehicle or 0.1 mg/kg of dictyostatin, or a biweekly dose of 0.3 mg/kg of dictyostatin (*n* = 11 for each group), for an intended study duration of 3 months. In addition, a cohort of age-matched non-Tg B6 male mice (*n* = 9) received vehicle injections once-weekly. At these lower dictyostatin doses, the body weight profile did not differ significantly between the compound- and vehicle-treated PS19 mice (Fig. 3), although all PS19 mice lost some weight over the course of the study in contrast to the wild-type controls, consistent with frailty in these mice that presumably results for tau transgene expression. Notably, 6 mice in the biweekly 0.3 mg/kg dictyostatin group and 3 mice in the weekly 0.1 mg/kg dictyostatin group died during the course of the study between weeks 9 and 13, although they did not show evidence of body weight loss that was significantly greater than the vehicle-treated PS19 mice. As with the PS19 mice treated with higher doses of dictyostatin, these mice also showed evidence of GI complications.

All of the PS19 mice surviving to the study completion underwent an analysis of major organ weight, and no significant differences were observed between any of the mice receiving either of the dictyostatin doses or the vehicle group (Table 1). Interestingly, no differences were observed in neutrophil counts or total white blood cell counts (nor red blood cell counts) in the dictyostatin treatment groups relative to the vehicle-treated PS19 mice (Table 2). As neutropenia is one of the major complications observed with high doses of MT-stabilizing agents used for the treatment of cancer [6, 18], these data suggest that peripheral drug exposure in the biweekly 0.3 mg/kg and weekly 0.1 mg/kg dictyostatin treatment groups was not sufficient to elicit this side effect. Although further studies would be required to fully understand the nature of the GI toxicity observed in the PS19 mice, we speculate that it may result from inhibition of mucosal epithelial cell division that has been suggested to be the cause of chemotherapy-induced enteropathy in patients receiving anti-cancer drugs [32], including MT-stabilizing taxanes [19].

**Table 1:** Comparison of organ weights (expressed as percentage of body weight) in PS19 mice treated for 14 weeks with vehicle, weekly doses of 0.1 mg/kg dictyostatin, or biweekly doses of 0.3 mg/kg dictyostatin

	Vehicle	Dicytostatin (0.3 mg/kg)	Dictyostatin (0.1 mg/kg)
Liver	5.34 +/−0.80	4.90 +/−0.56	5.36 +/−1.20
Spleen	0.28 +/−0.04	0.34 +/−0.07	0.29 +/−0.04
Kidney	1.79 +/−0.14	1.72 +/−0.16	1.65 +/−0.14
Heart	0.60 +/−0.05	0.58 +/−0.02	0.56 +/−0.03

**Table 2:** Comparison of blood cell counts in PS19 mice treated for 14 weeks with vehicle, weekly doses of 0.1 mg/kg dictyostatin, or biweekly doses of 0.3 mg/kg dictyostatin

	WBC (k/μl)	Neutrophils (#/μl)	RBC (M/μl)
Vehicle	1.99 +/−1.42	516 +/−438	7.81 +/−0.38
Dictyostatin (0.3 mg/kg)	2.74 +/−2.59	764 +/−436	7.92 +/−0.52
Dictyostatin (0.1 mg/kg)	1.86 +/−1.19	860 +/−724	7.60 +/−0.72

*WBC* white blood cells, *RBC* red blood cells

Due to the loss of over 50% of the 0.3 mg/kg dictyostatin cohort, efficacy analyses were not conducted with this treatment group due to the loss of statistical power. However, all of the 0.1 mg/kg dictyostatin-treated PS19 mice that persisted to study completion (*n* = 8), as well as the vehicle-treated PS19 and wild-type mice, underwent analyses of a number of CNS measures, as described below. No cognitive testing was attempted with the PS19 mice due to the decreases in group sizes and of concerns that underlying side effects might compromise testing outcomes.

### Dictyostatin improved brain MT density and reduced both axonal dystrophy and tau pathology in PS19 tau Tg mice, with a trend toward lessened hippocampal neuron loss

**Fig. 4:**
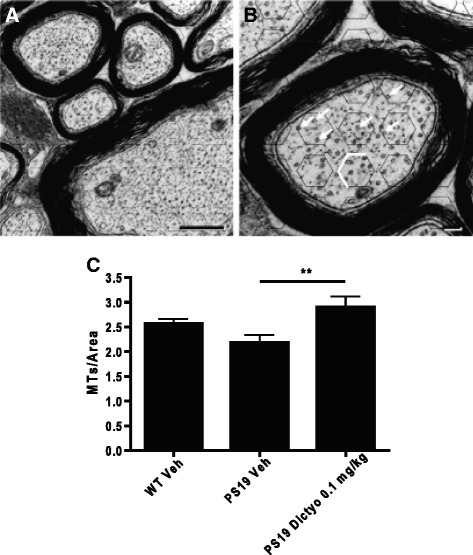
Dictyostatin treatment increased MT density in optic nerves of aged PS19 mice. MTs were counted in electron micrographs (50,000X) of cross-sections of optic nerves (**a** bar = 0.5 μm) from dictyostatin- or vehicle-treated aged PS19, or vehicle-treated WT mice, as previously described [46]. **b** An expanded imaged showing the grid of hexagons applied to optic nerve EM images (bar = 0.1 μm). To avoid repeat counting, only MTs that resided within the hexagon or were on one of the three indicated borders of each hexagon were counted. MTs are identified by arrows. **c** Quantification of MTs/area in the optic nerves of mice from each treatment group. Error bars denote SEM, with group sizes of 8–11. **, *p* < 0.01 as determined by one-way ANOVA with a post-hoc Tukey’s multiple comparison test

**Fig. 5:**
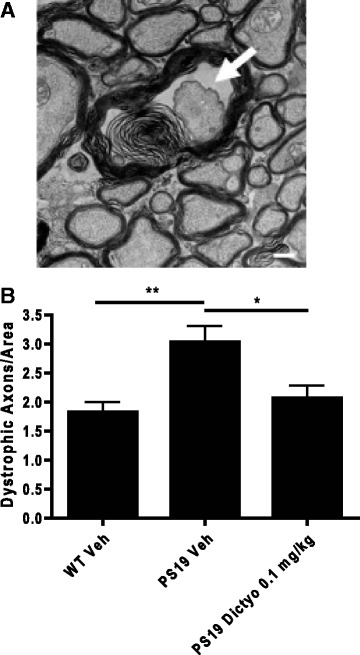
Dictyostatin treatment decreased axonal dystrophy in optic nerves of aged PS19 mice. **a** A representative electron micrograph showing an example of a dystrophic axon within the optic nerve from a study mouse (bar = 0.5 μm). **b** The number of dystrophic axons were counted in electron micrographs of cross-sections of optic nerves from dictyostatin- or vehicle-treated aged PS19 or WT mice, as previously described [46]. Error bars denote SEM, with group sizes of 8–11. *, *p* < 0.05; **, *p* < 0.01 as determined by one-way ANOVA with a post-hoc Tukey’s multiple comparison test

We demonstrated previously that aged PS19 mice have a modest deficit in MT density, as determined through a comparison of MT counts in cross-sectional electron microscopic images of optic nerve (ON) sections from non-Tg littermates and PS19 tau Tg mice [15, 46], which harbor tau pathology in retinal ganglia cells [15]. In addition to a small reduction in total MTs, tau Tg mice have also been shown to have an increase in MT dynamicity [5], again reflecting altered MT properties. Prior studies revealed that treatment with epothilone D increased MT density in the PS19 mice to levels that met or exceeded non-Tg mice of comparable age [15, 46]. A similar analysis was performed on the optic nerves with the PS19 mice from the 0.1 mg/kg dictyostatin and vehicle treatment groups (Fig. 4), as well as with vehicle-treated wild-type mice. As summarized in Fig. 4c, a small reduction of MT density was observed in the PS19 mice relative to age-matched non-Tg mice, and the PS19 mice that were treated with 0.1 mg/kg of dictyostatin showed a significant increase in MT numbers relative to the vehicle-treated PS19 mice, mimicking the effect previously observed with epothilone D [15, 46]. In the prior reported studies, the epothilone D-mediated improvement in MT density within tau tangle-bearing PS19 mice resulted in a significant decrease in the number of dystrophic retinal ganglion cell axons within the ON. This was also observed in the dictyostatin-treated PS19 mice, as axonal dystrophy (Fig. 5a) was significantly reduced compared to vehicle-treated PS19 mice, approaching the level observed in the non-Tg mice (Fig. 5b).

**Fig. 6:**
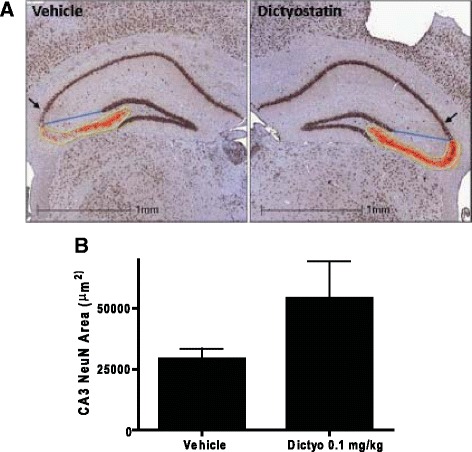
Dictyostatin treatment resulted in a trend towards reduced hippocampal CA3 neuron loss in aged PS19 mice. **a** Bregma-matched hemisphere brain sections from dictyostatin- or vehicle-treated aged PS19 mice (two sections/mouse) were immunostained with NeuN antibody and the area occupied by NeuN staining was determined within the demarcated CA3 region. The end boundary of the CA3 region was defined by drawing a line from the dentate to the start of the CA2 region (*blue line*), also using the demarcation of the end of the CA2 region (*black arrow*) as a landmark. Representative images with the quantified area are depicted. Scale bar = 1 mm. **b** A comparison of the results of NeuN staining in vehicle- and dictyostatin-treated PS19 mice (*n* = 7 for the dictyostatin group and *n* = 9 for the vehicle group). Error bars denote SEM

One feature of PS19 Tg mice is a loss of hippocampal neurons, particularly in the CA3 region, upon the development of significant tau pathology [45, 46]. To examine whether any differences were observed in hippocampal neuron density in the PS19 mice treated with 0.1 mg/kg dictyostatin compared to vehicle-treated mice, Bregma-matched fixed brain sections from each mouse were immunostained with NeuN antibody and the NeuN-positive area occupied in the CA3 region of the hippocampus was quantified. The PS19 mice treated with dictyostatin had an increase in hippocampal CA3 NeuN area compared to the vehicle-treated group (Fig. 6), although this did not reach statistical significance. The trend in these data, in which only 7 dictyostatin-treated mice were evaluated due to poorly preserved tissue in the brain sections from one animal, are consistent with prior reports of beneficial effects of MT-stabilizing agents on neuronal survival in tau Tg mice [5, 15, 46], and further suggest that the low 0.1 mg/kg dose of dictyostatin provided benefit to the brains of the aged PS19 mice.

**Fig. 7:**
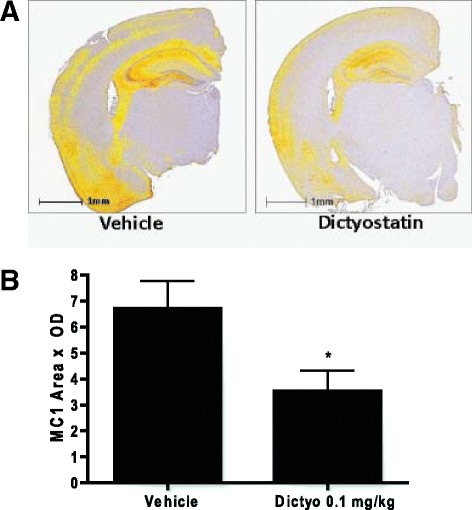
Dictyostatin treatment reduced MC1-positive tau pathology. **a** Bregma-matched hemisphere brain sections from dictyostatin- or vehicle-treated aged PS19 mice (two sections/mouse) were immunostained with MC1 antibody, and the total integrated MC1 signal over the section was obtained. Representative images with quantified areas and relative staining intensity are depicted, where the intensity of staining is shown as a yellow (low OD) to red (high OD) spectra. Scale bar = 1 mm. **b** A comparison of the results of MC1 staining in vehicle- and dictyostatin-treated PS19 mice (*n* = 7 for the dictyostatin group and *n* = 10 for the vehicle group). Error bars denote SEM. *, *p* < 0.05 as determined by a two-tailed *t*-test

**Fig. 8:**
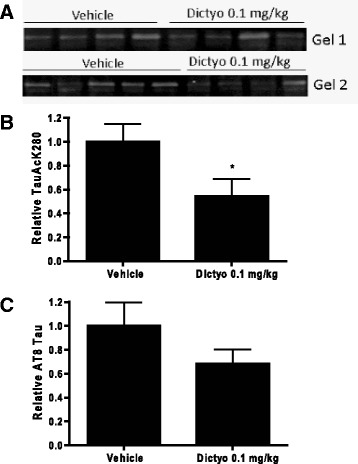
Dictyostatin treatment caused a significant reduction of insoluble TauAcK280. **a** The high salt buffer-insoluble fraction from combined cortical and hippocampal homogenates of vehicle- and dictyostatin-treated aged PS19 mice were analyzed for the amount of TauAcK280 by immunoblotting. Samples from PS19 mice receiving vehicle (*n* = 9) or 0.1 mg/kg of dicytostatin (*n* = 8) were analyzed on two separate gels, with the densitometric values for TauAcK280 bands from the vehicle and dictyostatin samples (**a**) compared and normalized for each gel after immunoblotting. **b** A plot of the relative TauAcK280 values in the vehicle- and dictyostatin-treated PS19 mice**. c** A similar analysis was performed in which immunoblots were probed with the AT8 antibody that recognizes tau that is phosphorylated at Ser202/Thr205. Error bars denote SEM. *, *p* < 0.05 as determined by a two-tailed *t*-test

Finally, we and others demonstrated that epothilone D-treated tau Tg mice have evidence of reduced tau pathological burden in their brains [5, 46]. An evaluation of tau pathology in Bregma-matched sections from the vehicle- and dictyostatin-treated PS19 mice, as determined after immunostaining with the MC1 antibody [31] that recognizes misfolded tau, revealed a significant reduction in pathologic tau in the dictyostatin treatment group (Fig. 7). To further confirm an effect of dictyostatin on pathological tau, the amount of insoluble tau that was acetylated at lysine residue 280 (TauAcK280) and phosphorylated at residues Ser202/Thr205 was quantified by fractionation of brain homogenates and subsequent immunoblot analysis of the high salt-insoluble fraction with TauAcK280 and AT8 antibodies, respectively. TauAcK280 is a marker of mature tau tangles [30], and the PS19 mice treated with 0.1 mg/kg of dictyostatin had significantly lower levels of insoluble TauAcK280 than did the PS19 cohort treated with vehicle (Fig. 8a, b). There was a similar trend toward reduced high salt-insoluble AT8-tau in the dicytostatin-treated group (Fig. 8c), although this difference did not reach statistical significance. Taken in totality, the immunohistochemical and immunoblot data indicate that dictyostatin treatment resulted in a reduction of tau pathology.

## Conclusion

In conclusion, 6-month old PS19 mice with existing tau pathology treated with a low dose of dictyostatin (0.1 mg/kg weekly) for 3 months demonstrated improvement in several CNS measures relative to vehicle-treated PS19 Tg littermates. In this regard, the results observed after dictyostatin treatment were generally comparable to those obtained previously with epothilone D. However, epothilone D is well tolerated at efficacy doses and above in PS19 Tg mice, whereas these mice showed evidence of intolerance to dictyostatin.

## Discussion

Tau is believed to play an important role in the regulation of neuronal axonal transport through its association with MTs. In addition to providing stabilization to MTs [22, 24], tau may also regulate the binding of kinesin and dynein to MTs [21, 41, 42], thereby affecting axonal transport. In neurodegenerative tauopathies, the hyperphosphorylation of tau results in disengagement from MTs such that MT dynamics and axonal transport are believed to be affected in ways that could contribute to the tau pathology and neuronal degeneration observed in these diseases. In this regard, there is evidence of MT deficits in the AD brain [16, 26], as well as in Tg mouse models of tauopathy [5, 15, 46, 47]. This led our laboratories to investigate the potential utility of small molecule MT-stabilizing agents for the treatment of tauopathies. Importantly, we demonstrated previously that the brain-penetrant MT-stabilizing small molecule, epothilone D, had salutary effects in both preventative [15] and interventional [46] studies with PS19 tau Tg mice that develop extensive tau pathology and neuron loss with associated memory impairment. The benefits of epothilone D included improved MT density, reduced axonal dystrophy, increased axonal transport and, importantly, reductions in both neuron loss and tau pathology. In addition, the beneficial effects of epothilone D were associated with improved cognitive performance in the PS19 mice, which is significant since clinical improvement is a requirement for approval of a drug for AD and related tauopathies. Moreover, these benefits were obtained with a dose of epothilone D that was ~1/100^th^ the human equivalent dose used previously in clinical trials in cancer patients [7], without evidence of any observable side effects. These positive effects of epothilone D were confirmed in two additional tau Tg mouse models [5], where this MT-stabilizing agent was also shown to mitigate the MT hyperdynamicity observed in the tauopathy mice.

Subsequent to the publication of these studies, epothilone D (BMS-241027) was tested in a short 9-week trial in AD patients (ClinicalTrials.gov identifier: NCT01492374) at doses that were roughly comparable to those used in the aforementioned mouse studies, when adjusted for species. Although the results of this small Phase 1b study have not been published, there has been no additional reported testing of epothilone D. More recently, a MT-stabilizing agent from the taxane family, TPI-287, has entered clinical testing in patients with AD (ClinicalTrials.gov identifier: NCT01966666) and PSP/CBD (ClinicalTrials.gov identifier: CT02133846). To date, there are no publications on the efficacy of this drug in mouse models of tauopathy, and the clinical studies with TPI-287 were designed to be 9 weeks in duration. Thus, it will be of interest to see whether changes in disease biomarkers can be observed after this relatively brief dosing period, which is much shorter than the typical 18–24 month AD efficacy trials. Finally, the octapeptide davunetide, which has reported MT-stabilizing activity [20] and which improved outcomes in tau Tg mice [35, 40], underwent testing in a 1-year Phase 2/3 trial in PSP patients in which the drug was administered intranasally. Unfortunately, the study results indicated that davunetide was not an effective treatment for PSP [8].

As the nasally-administered peptide davunetide is the only MT-stabilizing agent that has undergone examination in an extended tauopathy clinical trial, there is merit in identifying and testing new examples that might serve as drug candidates. Herein, we have examined the effects of dictyostatin in a 3-month interventional study utilizing 6-month old PS19 tau Tg mice that harbor a low level of existing tau pathology which progresses to become more pronounced as the mice reach 9 months of age. The original intent was to treat the PS19 mice with weekly doses of 0.3 mg/kg and 1.0 mg/kg of dictyostatin, but these doses were poorly tolerated by the mice, with evidence of GI morbidities. Additional studies are needed to gain a greater understanding of this GI complication, but we speculate that dividing intestinal epithelial cells may be affected by the MT-stabilizing agent, as has been reported with anti-cancer drugs in some oncology patients [32], including those administered MT-stabilizing taxanes [19].

In response to the GI complications elicited by the weekly doses of 1.0 and 0.3 mg/kg of dictyostatin, we initiated a subsequent study in which the compound was administered biweekly at 0.3 mg/kg, or weekly at a lower dose of 0.1 mg/kg. Although these dosing regimens did not lead to weight loss that was distinguishable from PS19 mice receiving vehicle, a significant proportion of the mice in the 0.3 mg/kg dose group and some mice in the 0.1 mg/kg dose group died 9–13 weeks into the study. Notably, neutropenia, which is one of the major side effects in cancer patients taking MT-stabilizing drugs, was not observed in the PS19 mice receiving either dictyostatin dose.

As the majority of the PS19 mice receiving weekly doses of 0.1 mg/kg dictyostatin completed the study, CNS outcome measures were compared in this treatment group and in vehicle-treated PS19 mice, recognizing that statistical power was reduced due to the loss of some mice in the dicytostatin group. The PS19 mice receiving the low dose of dictyostatin showed marked improvements in several efficacy measures. In particular, dictyostatin improved MT density with a clear reduction of axonal dystrophy, resembling the beneficial effects previously observed with epothilone D [15, 46]. Similarly, dicytostatin treatment led to a trend towards reduced hippocampal CA3 neuron loss, and a significant reduction of MC1- and AcK280-positive tau pathology. Again, these improvements were similar to those observed with epothilone D, indicating that dictyostatin has comparable effects to epothilone D in improving CNS/brain outcomes in this mouse model of tauopathy.

These data provide additional support for the potential clinical utility of small molecule MT-stabilizing agents for the treatment of tauopathies, but also reinforce the importance of achieving efficacy at drug doses that are well tolerated. We have previously speculated that the excellent safety profile of epothilone D may result from the long retention of the drug in the brain and the relatively rapid clearance from blood, such that peripheral exposures are minimized while sufficient brain concentrations are maintained to provide prolonged benefit [13]. In this regard, dictyostatin shows similarities to epothilone D, with relatively rapid clearance from blood with retention in the brain [11]. Thus, the differences in the toxicity profiles between these two MT-stabilizing agents was unexpected and may relate to differential retention in one or more peripheral sites, for example within intestinal mucosa cells. Alternatively, the disparities in the safety profiles of epothilone D and dictyostatin may result from differing binding affinities to MTs. We have demonstrated that dictyostatin is more potent than epothilone D in a cellular assay of MT-stabilization [11], and the fact that a very low dose (0.1 mg/kg) of dictyostatin provided MT stabilization in the brain could reflect a tight association/slow dissociation from MTs that also increases the chance for peripheral side effects. Finally, we cannot exclude the possibility that the GI complications observed upon dictyostatin dosing are due to a drug metabolite, or to an off-target interaction unrelated to MT stabilization. Moreover, it is unclear whether a similar side effect profile would be observed in humans upon comparable exposure to dictyostatin, as peripheral neuropathy and neutropenia are the most common dose-limiting toxicities observed after treatment with MT-stabilizing drugs [6, 17, 18] and neutropenia was not observed at the efficacy dose of dictyostatin. Thus, it is possible that dictyostatin may still have potential as a candidate for human tauopathies, although the results from our studies indicate that additional safety studies would be required to gain a greater understanding of the GI complications observed in PS19 tau Tg mice.

## Abbreviations

AD: Alzheimer’s disease
CBD: Cortibasal degeneration
CNS: Central nervous system
FTLD: Frontotemporal lobar degeneration
GI: Gastrointestinal
MT: Microtubule
ON: Optic nerve
PSP: Progressive supranuclear palsy
Tg: Transgenic
